# Bacterial DNA metabolism analysis by metagenomic next-generation sequencing (mNGS) after treatment of bloodstream infection

**DOI:** 10.1186/s12879-023-08378-7

**Published:** 2023-06-12

**Authors:** Zhuo Li, Shiying Zhang, Mengting Liu, Hongguang Ding, Yin Wen, Huishan Zhu, Hongke Zeng

**Affiliations:** 1 Department of Critical Care Medicine, Guangdong Provincial People’s Hospital (Guangdong Academy of Medical Sciences), Southern Medical University, Guangzhou, Guangdong, 510080 China; 2 Department of Emergency, Guangdong Provincial People’s Hospital (Guangdong Academy of Medical Sciences), Southern Medical University, Guangzhou, Guangdong, 510080 China; 3 Department of Critical Care Medicine, Zhujiang Hospital, Southern Medical University, Guangzhou, Guangdong, 510280 China

**Keywords:** mNGS, Bloodstream infection, DNA metabolism

## Abstract

**Background:**

With the advent of metagenomic next-generation sequencing (mNGS), the time of DNA metabolism can be explored after bacteria be killed. In this study, we applied mNGS in investigation of the clearance profile of circulating bacteria DNA.

**Methods:**

All of the rabbits were injected with the inactivated Escherichia coli. Using mNGS, we analyzed serial samples of plasma collected from rabbits to detect clearance profile of circulating *E. coli* DNA.

**Results:**

In this study, we found that the of *E. coli* DNA could still be detected 6 h after injecting killed bacteria. The clearance half-lives associated with the 2 phases are 0.37 and 1.81 h. We also explored there is no correlation between the disease severity with the *E. coli* DNA reads in circulation.

**Conclusions:**

After the bacteria were completely killed, their DNA could still be detected in the blood circulation. The metabolism of bacterial DNA in the circulation had two phases: fast and slow phases, and there were no correlations between the level of bacteria reads with the severity of patients’ disease after the bacteria have been completely killed.

**Supplementary Information:**

The online version contains supplementary material available at 10.1186/s12879-023-08378-7.

## Introduction

Bloodstream infections are associated with significant mortality. Early effective antimicrobial therapy has been demonstrated to improve patient outcomes. Escherichia coli (*E. coli*) bloodstream infection was considered a considerable and growing burden in human medicine [1]. Three studies reported 30-day case fatality risk between 8 and 18.2% for *E. coli* BSIs [2–4]. Additionally, due to the global emergence of multidrug-resistant (MDR) strains, the community antibiotic resistance in *E. coli* has increased dramatically, which may result from prolonged empirical antibiotic therapy [5].

In the clinical treatment of bloodstream infections, rapid diagnosis and initial treatment are necessary. Since 2010, metagenomic next-generation sequencing (mNGS) technology, based on nucleic acid sequencing to rapidly detect pathogens has been clinically applied and validated as a diagnostic method for infectious diseases [6, 7]. However, the timing of discontinuation after treatment of patients is difficult to determine clinically, which can lead to unnecessary antibiotic exposure. Therefore, adjustments should be made as soon as possible according to the microbiological spectrum [8, 9].

There have been reports Fetal DNA remains in the maternal circulation after birth, and there were no correlations between birth weight or number of fetal nucleated cells and absolute and fractional concentrations of fetal DNA [10, 11]. We guess that bacterial DNA remains in the patient’s circulation after the bacteria have been completely killed. It has important reference significance for the clinical selection of detection methods and drugs for pathogens.

Recent studies have begun to reveal the potential pro-inflammatory role of extracellular DNA (e-DNA) released by activated airway neutrophils in asthma due to the high concentration of e-DNA in sputum associated with elevated sputum neutrophils, activation of innate immune responses, and elevated sputum cytokines [12, 13]. However, whether the release of bacterial DNA from the inactivated bacteria will cause the aggravation of inflammation.

In this study, we aim to explore when the DNA of bacteria completely disappears after the pathogenic bacteria being killed and whether it is related to the disease severity, so as to provide a new idea for the clinical treatment of patients with bloodstream infection.

## Materials and methods

### Animals

Adult New Zealand rabbits (male, 2.0-2.5 kg) were purchased from Guangdong Medical Laboratory Animal Center. The rabbits were reared in experimental conditions (temperature, 20–25 °C; humidity, 50–70%) with a 12 h light/dark cycle and fed standard chow and water. All of the rabbits were injected with the inactivated *E. coli*.

### Bacteria

The parental strain used in this study was the drug-susceptible *E. coli* ATCC 25,922. All other cultures were derivatives produced by chemostatic culture. The lyophilized strains were resuscitated on fresh Lennox AGAR (Sigma-Aldrich) and the growth of individual colonies was transferred to lysogenetic broths (LB; Sigma-Aldrich). In order to ensure that the parental strain utilized throughout the continuous culturing was genetically homogeneous at the outset, this single colony suspension was used to inoculate three parallel chemostat vessels. The continuous cultures were grown in quarter-strength LB medium to ensure nutrient restriction. The developed cultures were stored at − 20 °C in 20% glycerol.

Concentration detection: The dilution plate coating counting method and blood cell counting plate were used to count the number of bacteria and UV spectrophotometry was used to measure OD value of bacterial culture medium, and a standard curve of concentration-OD value was formed for measuring the concentration of bacterial suspension.

### Animal model of *E. coli* bloodstream infection

Rabbits were anesthetized by intraperitoneal injection of 3% sodium pentobarbital (60 mg/kg). Indwelling trocar was inserted into the auricular vein for fixation. The model of bloodstream infection was established by intravenous injection of 1ml inactivated 1*10^8^ CFU/mL Escherichia coli suspension.

### Sample collection and preparation

Two milliliters of peripheral blood were collected from each auricular vein in Rabbit. Two milliliters of blood were collected into EDTA-containing tubes: 0.5 h, 1.5 h, 2 h, 3 h, and 6 h after the inactivated *E. coli* was injected and centrifuged at 1500 r/min for 5 min, and the plasma was collected.

### DNA sequencing

#### Library preparation and metagenomic sequencing

DNA library was prepared by automatic nucleic acid extraction, enzymatic fragmentation, end repair, terminal adenylation, and adaptor ligation according to a previous study. Finished libraries were quantified by real-time PCR (KAPA) and pooled. Shotgun sequencing was carried out on illumina Nextseq. Approximately 20 million of 50 bp single-end reads were generated for each library. Bioinformatic analysis was conducted as described in a previous report. Briefly, sequences of human origin were filtered (GRCh38.p13) and the remaining reads were aligned to a reference database (NCBI nt, GenBank, and in-house curated genomic database) to identify the microbial species and read count. For each sequencing run, a negative control (culture medium containing 10^4^ Jurkat cells/mL) was included.

#### mNGS reporting criteria

Microbial reads identified from a library were reported if: (1) the sequencing data passed quality control filters (library concentration > 50 pM, Q20 > 85%, Q30 > 80%); (2) negative control (NC) in the same sequencing run does not contain the species or the RPM (sample) / RPM (NC) ≥ 5, which was determined empirically according to previous studies as a cutoff for discriminating true-positives from background contaminations [14].

### Elisa test

Interleukin-6 (IL-6), procalcitonin (PCT) expression in the plasma of rabbits were quantified by an ELISA kit (R&D Systems, Inc., Minneapolis, MN, USA). Plasma was collected from the peripheral blood at 1.5 h, 3 h, and 6 h after killed *E. coli* was injected and quantified for IL-6, PCT expression using the ELISA kit, following the manufacturer’s instructions.

### Statistical analysis

Statistical significance was determined using t test with *P* < 0.05 considered to be significant. Correlation studies were performed using Pearson correlation coefficient.

## Results

### Detection of *E. coli* DNA in plasma of rabbits injected with inactivated *E. coli*

In order to detect when the DNA of the bacteria completely disappeared after being completely killed, we used to inject the rabbits with heated inactivated *E. coli* to simulate the blood flow infection model of the rabbits. In total, we analyzed 21 plasma samples from 3 rabbits. Blood samples were collected at 0.5 h, 1.5 h, 2 h, 3 h, 6 and 48 h after modeling, and the reads of *E. coli* DNA in the plasma of rabbits were detected (Table 1). Therefore, circulating *E. coli* DNA in rabbits disappeared by 1 to 2 days after *E. coli* has been completely eliminated.

**Table 1:** Reads in plasma from New Zealand rabbit after injection of killed E. coli

Sample Collection time	E. coli DNA Reads				
	Sample1	Sample2	Sample3	mean	Std. Error of Mean
0.5 h	22,325	14,821	12,386	16,511	2990.94
1.5 h	2661	2145	2436	2414	149.36
2 h	455	446	506	469	18.68
3 h	387	260	280	309	39.43
6 h	109	67	136	104	20.07
48 h	0	0	0	0	0

### Clearance kinetics of circulating *E. coli* DNA

**Fig. 1:**
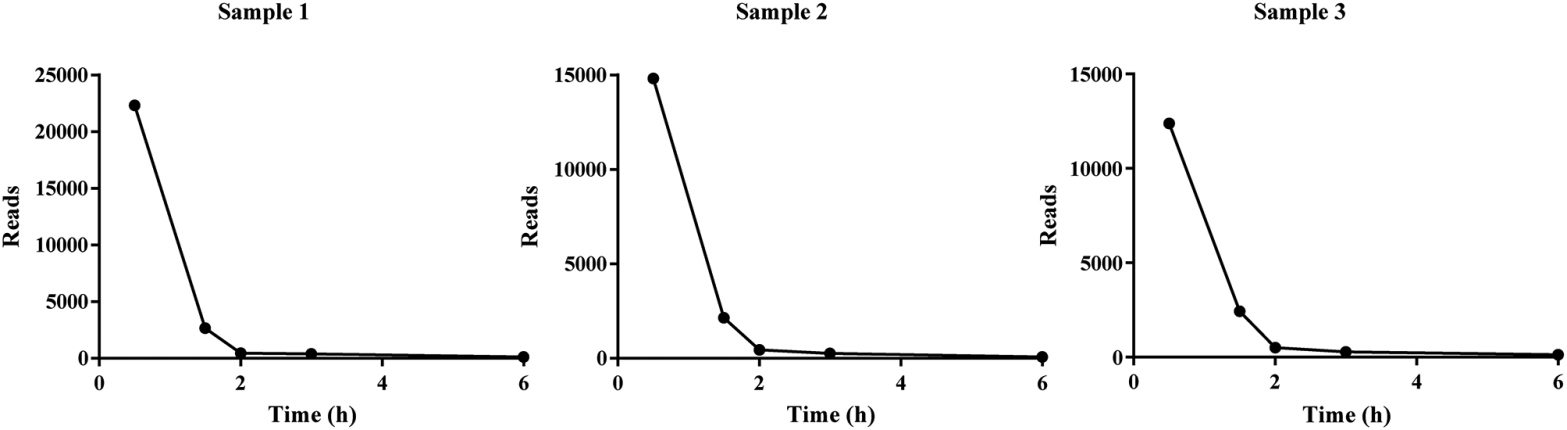
Dynamics of *E. coli* DNA clearance. Sample1-3(within 6 h after the bacteria were completely killed). Time is plotted on the x axis, and the reads of *E. coli* is plotted on the y axis

We investigated the clearance kinetics of circulating *E. coli* DNA by determining the absolute *E. coli* DNA reads of each rabbit plasma sample measured by mNGS. The clearance profiles for the absolute *E. coli* DNA concentrations were similar for samples 1–3 (Fig. 1). *E. coli* DNA was also detectable in New Zealand rabbit plasma after *E. coli* has been completely killed.

**Fig. 2:**
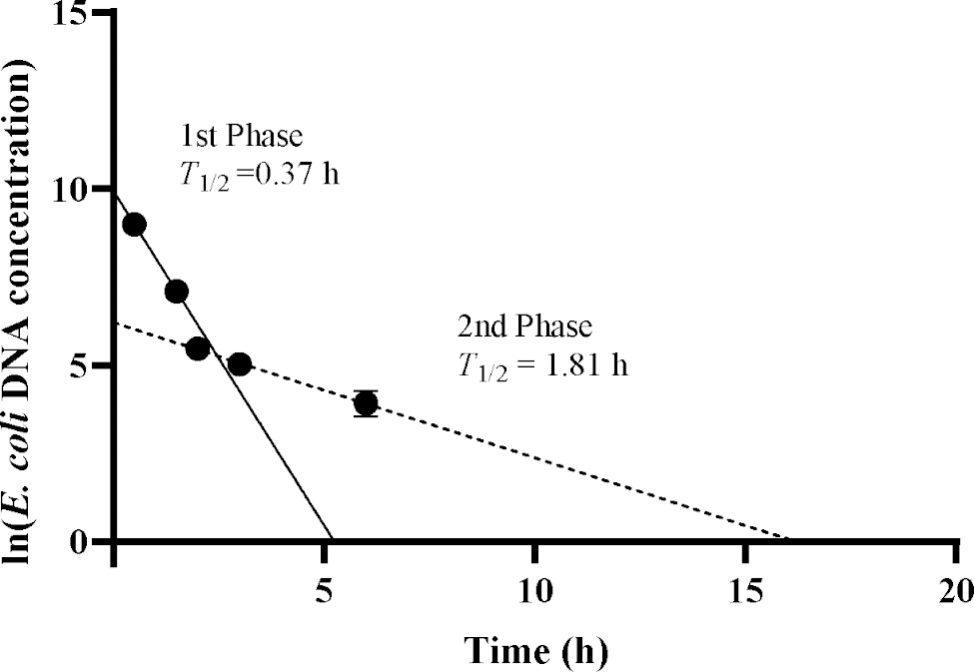
Clearance kinetics of circulating *E. coli* DNA. Time is plotted on the x axis, and the reads of *E. coli* is plotted on the y axis. Linear regression lines for the rapid phase and the slow phase are represented by solid and dashed lines, respectively. The corresponding half-lives (T1/2) for the 2 phases are shown

Plotting the natural logarithm of the absolute *E. coli* DNA concentration against time demonstrated that all samples had a 2-phase clearance pattern—a rapid initial phase followed by a slow phase (Fig. 2). For all samples, we also determined the slopes of the plots and calculated the half-lives with the following equation: Half-life = ln (2)/Slope. The mean half-lives of the rapid phase (0–2 h after) and the slow phase (3–6 h after) were 0.37 and 1.81 h respectively.

### Correlations of the severity of disease with circulating concentration of *E. coli* DNA

**Fig. 3:**
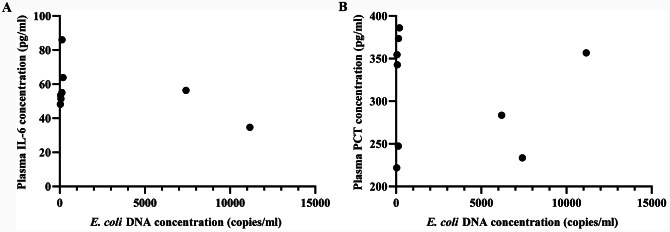
The relationship between the IL-6 and PCT with circulating concentration of *E. coli* DNA

We explored whether the severity of the disease of the New Zealand rabbits was correlated with the circulating reads of *E. coli* in the samples of New Zealand rabbit plasma. No significant Spearman correlations were found among the circulating reads of *E. coli* and IL-6 (Fig. 3A) (*r* = -0.510, *P* = 0.187), PCT (Fig. 3B) (*r* = -0.100, *P* = 0.798).

## Discussion

This work represents use the bacterial DNA sequences in the plasma of rabbits for studying the clearance profile of circulating bacterial DNA. We found that the of *E. coli* DNA could still be detected 6 h after injecting killed bacteria. The clearance half-lives associated with the 2 phases are 0.37 and 1.81 h. We also explored there is no correlation between the disease severity with the *E. coli* DNA reads in circulation.

Stephanie C.Y. Yu et al. previously reported that in maternal plasma collected from 13 women after cesarean delivery the final disappearance of circulating fetal DNA occurred at about 1 to 2 days postpartum [10]. Similarly, *E. coli* DNA could still be detected in the peripheral blood of rabbits after intervention. The results showed that the detection of pathogenic bacteria DNA in the plasma of patients after clinical treatment does not mean that there are still live bacteria in the body. After the bacteria have been completely killed, but the residual bacterial DNA has not been completely eliminated. It is recommended to test again after 6 h to determine the presence of live bacteria at the initial test.

Y. M. Dennis Lo et al. analyzed serial samples of maternal plasma collected at multiple time points within 2 h postpartum to explore the characteristics of fetal DNA metabolism in the maternal plasma after delivery [15]. The present study extended the serial blood sampling to 48 h after we injected them with heated inactivated Escherichia coli in 3 samples. Results from these 3 samples suggest that circulating *E. coli* DNA is cleared in 2 phases. The clearance half-lives of the two phases were 0.37 and 1.81 h, respectively. We speculate that this biphasic clearance pattern of circulating *E. coli* DNA may have implications for the underlying mechanisms of DNA removal.

Previous study noted that infected patients experienced a transient increase in inflammatory response after antibiotic treatment, but endotoxin expression was not elevated [16]. We speculate that bacteria killed by antibiotics, their DNA is released into the bloodstream in large quantities. Whether there were correlations between the severity of the disease with circulating concentration of *E. coli* DNA. But as the results turned out, they were unrelated. Therefore, we can get inspiration that after systematic treatment, the high reads of bacterial DNA do not mean the patient’s condition is serious, which has important reference significance for the clinical treatment of patients with bloodstream infection.

There are 2 limitations in this study. In the present study, due to limited funds, the sample size of our test is relatively small. Additionally, the mechanisms of the two phases leading to rapid and slow bacterial DNA metabolism can be further studied.

## Conclusion

After the bacteria were completely killed, their DNA could still be detected in the blood circulation. The metabolism of bacterial DNA in the circulation had two phases: fast and slow phases, and there were no correlations between the level of bacteria reads with the severity of patients’ disease after the bacteria have been completely killed.

## Electronic supplementary material

Below is the link to the electronic supplementary material.

**Table S2**. The Pearson correlation between the *E. coli* DNA concentration and plasma inflammatory cytokine concentration

**Table S1**. Clearance half-life of circulating *E. coli* DNA

## Data Availability

The sequencing data reported in this study was archived in the Sequnece Read Archive (SRA) with the accession number PRJNA940603, with web link https://submit.ncbi.nlm.nih.gov/subs/sra/.
