# Supplementary material for: Bacterial DNA metabolism analysis by metagenomic next-generation sequencing (mNGS) after treatment of bloodstream infection
Source: BMC Infect Dis. 2023 Jun 12;23:392. doi: 10.1186/s12879-023-08378-7 (PMC10258974; doi:10.1186/s12879-023-08378-7)
Supplement: Supplementary file 1 — Table S2. The Pearson correlation between the E. coli DNA concentration and plasma inflammatory cytokine concentration [file 12879_2023_8378_MOESM1_ESM.docx]

| **Table S2. The Pearson correlation between the E. coli DNA concentration and plasma inflammatory cytokine concentration** | | |
| --- | --- | --- |
|  | r^a^ | *P* value |
| IL-6 | -0.510 | 0.187 |
| PCT | -0.100 | 0.798 |
| TNF-α | 1.000 | 0.000^**^ |

^a^ Correlation coefficient.

^**^ Correlation is significant at the 0.01 level.
