# Supplementary material for: Bacterial DNA metabolism analysis by metagenomic next-generation sequencing (mNGS) after treatment of bloodstream infection
Source: BMC Infect Dis. 2023 Jun 12;23:392. doi: 10.1186/s12879-023-08378-7 (PMC10258974; doi:10.1186/s12879-023-08378-7)
Supplement: Supplementary file 2 — Table S1. Clearance half-life of circulating E. coli DNA [file 12879_2023_8378_MOESM2_ESM.docx]

| Table.S1 Clearance half-life of circulating *E. coli* DNA | | |
| --- | --- | --- |
|  | Half-life (hour)^a^ | ^b^R^2^ |
| 0-1 h | 0.37 | 0.963 |
| 2-6 h | 1.81 | 0.913 |

^a^ Half-life = − ln (2) / slope, slope of the plot of the natural logarithm of the *E. coli* DNA concentration against time in hour.

^b^ R^2^, coefficient of determination of a linear regression.
